# Behavioral and psychosocial factors related to mental distress among medical students

**DOI:** 10.3389/fpubh.2023.1225254

**Published:** 2023-07-27

**Authors:** Kathleen M. Carlos, Hedyeh Ahmadi, Kristina A. Uban, Jenna L. Riis

**Affiliations:** ^1^Program in Public Health, Department of Health, Society, and Behavior, University of California, Irvine, Irvine, CA, United States; ^2^Institute for Interdisciplinary Salivary Bioscience Research, University of California, Irvine, Irvine, CA, United States; ^3^Developing Brains Laboratory, Program in Public Health, Department of Health, Society, and Behavior, University of California, Irvine, Irvine, CA, United States; ^4^Department of Psychological Science, University of California, Irvine, Irvine, CA, United States

**Keywords:** depression, suicidal ideation, suicide, sleep, impostor syndrome, stress, medical students, financial distress

## Abstract

**Introduction:**

Physicians die by suicide at rates higher than the general population, with the increased risk beginning in medical school. To better understand why, this study examined the prevalence of mental distress (e.g., depressive symptoms and suicide risk) and behavioral and psychosocial risk factors for distress, as well as the associations between mental distress and risk factors among a sample of medical students in a pre–COVID-19-era.

**Methods:**

Students enrolled in a large California medical school in 2018–2019 (*N* = 134; 52% female) completed questionnaires assessing sociodemographic characteristics, depression and suicide family history, health behaviors, and psychosocial wellbeing. Assessment scores indexing mental distress (e.g., depressive symptoms, thoughts of suicide in the past 12 months, suicide risk, and history of suicidality) and risk factors (e.g., stress, subjective sleep quality, alcohol use, impostor feelings, and bill payment difficulty) were compared across biological sex using chi-squared tests, and associations between mental distress and risk factors were determined through logistic regression.

**Results:**

Elevated mental distress indicators were observed relative to the general public (e.g., 16% positive depression screen, 17% thought about suicide in previous 12 months, 10% positive suicide risk screen, and 34% history of suicidality), as well as elevated risk factors [e.g., 55% moderate or high stress, 95% at least moderate impostor feelings, 59% poor sleep quality, 50% screened positive for hazardous drinking (more likely in females), and 25% difficulty paying bills]. A positive depression screen was associated with higher stress, higher impostor feelings, poorer sleep quality, and difficulty paying bills. Suicidal ideation in the previous 12 months, suicide risk, and a history of suicidality were independently associated with higher levels of impostor feelings.

**Discussion:**

Higher scores on assessments of depressive symptoms and suicidal thoughts and behaviors were related to several individual-level and potentially modifiable risk factors (e.g., stress, impostor feelings, sleep quality, and bill payment difficulties). Future research is needed to inform customized screening and resources for the wellbeing of the medical community. However, it is likely that the modification of individual-level risk factors is limited by the larger medical culture and systems, suggesting that successful interventions mitigate suicide risk for medical providers need to address multiple socio-ecological levels.

## 1. Introduction

The COVID-19 pandemic brought the suffering of healthcare workers into sharp focus (1). Although it is still early to speculate, emerging research suggests that the pandemic likely exacerbated an issue that has been a topic of conversation in the peer-reviewed literature for decades (2).

Even prior to the pandemic, physicians suffered an increased risk of depression and suicide compared to the general population (3, 4), and research suggests that this increased risk begins in medical school. Before beginning medical school, matriculating medical students have higher self-reported quality of life and similar rates of depression compared to the general population (5), but this trend does not last. Medical students have a higher prevalence of depression and thoughts of suicide (i.e., suicidal ideation) than both the age-matched population and graduate students in other disciplines (6–9). A large nationwide study found that 58.2% of medical students screened positive for depression and 9.4% endorsed suicidal ideation (SI) within the previous 12 months (6). These rates are much higher than rates of major depression and SI reported by the general population [7.7–13.1 and 4.3–10.5%, respectively (9, 10)]. This shift in mental health during medical training highlights the need for additional research into the specific modifiable risk factors for depression and suicide (e.g., SI, suicide risk, suicidal thoughts, and behaviors) among medical students. Despite the extra attention on mental health that the COVID-19 pandemic has provided, a deep dive into modifiable risk factors for the development of depression and suicidality in this population has yet to be done. This study addresses that gap by examining risk factors in a pre–COVID-19 sample of medical students.

In this study, we concisely refer to depression and suicidality as mental distress, which is a broad and flexible term that encompasses self-reported mental health problems and the individual's experience of these problems. Depression and suicidality can be individually conceptualized as types of mental distress, as they are both mental health problems that individuals respond to uniquely and do not necessarily reflect diagnoses. There are many evidence-based risk factors for depression and suicidality in the general population; however, there is a specific gap in understanding the prevalence of these mental health conditions and their risk factors, and how they are associated among medical student populations. Previous studies have highlighted factors that place medical students at increased risk for depression including female gender (11, 12), burnout (6), lower emotional and mental quality of life (5), academic stress (13), perfectionism (11), stressful life events (11), long work hours (14), and lack of social support (15, 16). Factors that place medical students at increased risk for suicidal ideation have a large degree of overlap with risk factors for depression [i.e., female gender (17), burnout (7), and lower mental quality of life (7)]. Risk factors that are unique to suicidal ideation include dissatisfaction with academic performance (18), substance use (17, 18), being in the clinical years of training (12, 18), and demanding parents (17, 18). The purpose of the present study was to focus on risk factors that could be modifiable at the individual level. This study examined associations between indicators of mental distress [including depressive symptoms, SI, suicide risk, and history of suicidality (i.e., suicidal thoughts and behaviors)] and psychological, financial, and behavioral factors that often contribute to mental distress in the general population [including sleep quality (19–21), stress (22, 23), alcohol use (24, 25), feelings of impostor syndrome (26, 27), and financial distress (28, 29)] among a pre–COVID-19 sample of medical students in their 1st to 4th year of training. While these risk factors have demonstrated associations with mental wellbeing and are prevalent among medical school students, limited investigation has been dedicated to examining their relations with both depression and suicidality among medical students in the US. We also assess whether relations between the risk factors and mental distress indices remain after controlling for individual characteristics, such as biological sex, year in medical school, race, ethnicity, and family history of depression and suicide. Furthermore, we examine whether relations between the risk factors and suicidal risk remain after controlling for depressive symptoms. *A priori* hypotheses included predicted higher depressive symptoms and SI in our sample than typically reported in the general population, as well as predicted high levels of self-reported poor sleep quality, perceived stress, alcohol use, and feelings of impostor syndrome. We hypothesized that poor sleep quality, high perceived stress, high feelings of impostor syndrome, high alcohol use, and financial distress were associated with a positive depression screen, SI, and suicide risk. We also expected to see elevated risk factors among those with a history of suicidality. Given the estimated 3.25-fold higher rates of physician suicide among females compared to males (3), mental distress and risk factors were characterized by biological sex, predicting female students to report higher depression, suicidality, and feelings of impostor syndrome relative to male students. Similar rates of perceived stress, sleep quality, alcohol use, and financial distress were expected between male and female students.

## 2. Materials and methods

### 2.1. Study design and participants

All students actively enrolled full-time in a large, southern California medical school from December 2018 to August 2019 were eligible to participate in this cross-sectional study. The only exclusionary criterion was the inability to read English, but this did not exclude any potential participants. Permission was granted by the School of Medicine administration to contact the students through class email distribution lists. Participants were recruited primarily through email announcements sent by the primary author through a password-protected email account that was created for study use and that was only accessible by the primary author. Participants were also recruited through two in-person visits during times when the 3rd- and 4th-year (i.e., clinical stage) students were all in the same room for a required lecture. Students were not approached or emailed on an individual basis during recruitment. Participants who responded to the email announcements, or who emailed the study email after an in-person recruitment visit, were given additional study information, and an in-person appointment was scheduled to obtain informed consent and to fill out the pen-and-paper survey. The survey contained questions about sociodemographic characteristics, family history of depression and suicide, and validated measures of health behaviors and psychosocial wellbeing. Study participation was voluntary, and all survey responses were anonymized. Participants were immediately compensated $25 in cash for their time upon the completion of the survey. Study and recruitment procedures and materials were approved by the University's Institutional Review Board prior to recruitment.

### 2.2. Measures

#### 2.2.1. Depressive symptoms and depression screen

The Patient Health Questionnaire-9 (PHQ-9) was used to assess depressive symptom severity in the past 2 weeks (30). The PHQ-9 has good reliability and has been validated for use in student and general population samples (31). PHQ-9 scores range from 0 to 27 with a score of 10 used as a threshold for identifying individuals experiencing major depressive disorder with 88% sensitivity and 88% specificity (32, 33). This threshold was used to dichotomize PHQ-9 scores to indicate participants with a positive depression screen (DS; PHQ-9 score ≥10) vs. negative DS (PHQ-9 score <10).

#### 2.2.2. Suicidality

The Suicidal Behaviors Questionnaire-Revised (SBQ-R) was used to assess three unique indicators of suicidality: SI in the previous 12 months, lifetime history of suicidality, and overall suicide risk based on global scores. The SBQ-R is validated for use in college students and in the general population with good reliability (34). Scores range from 3 to 18 with a cutoff score of 7 having optimal sensitivity (93%) and specificity (95%) to differentiate between non-suicidal and suicidal individuals (34). This threshold was used to dichotomize the suicide risk variable. Due to the distribution of responses, lifetime history of suicidality and SI in the previous 12 months were also dichotomized based on participant answers to items 1 and 2 on the SBQ-R. Item 1 asks, “Have you ever thought about or attempted to kill yourself?” The answer choices include, “Never,” “It was just a brief passing thought,” “I have had a plan at least once to kill myself but did not try to do it,” “I have had a plan at least once to kill myself and really wanted to die,” “I have attempted to kill myself, but did not want to die,” and “I have attempted to kill myself, and really hoped to die.” Item 2 asks, “How often have you thought about killing yourself in the past year?” The answer choices include, “Never,” “Rarely (1 time),” “Sometimes (2 times),” “Often (3–4 times),” and “Very often (5 or more times).” If the participant answered “Never” to item 1, they were said to have no history of suicidality. All other responses to item 1 were coded as a history of suicidality. This same process was used for item 2, to determine the presence or absence of SI in the previous 12 months.

#### 2.2.3. Sleep quality

The Pittsburgh Sleep Quality Index (PSQI) was used to assess participants' perceived sleep quality over the past 30 days. PSQI scores range from 0 to 21 with higher scores indicative of worse sleep. The PSQI has been validated in clinical and non-clinical populations (35). A score ≥5 differentiates poor sleep quality with 89.6% sensitivity and 86.5% specificity (36). PSQI sum scores were dichotomized using this threshold.

#### 2.2.4. Alcohol use

The Alcohol Use Disorders Identification Test-C (AUDIT-C) was used to assess hazardous drinking and/or active alcohol use disorders (37). AUDIT-C scores range from 0 to 12, with scores ≥4 indicative of a positive screen for men and scores ≥3 of a positive screen for women (37). The recommended cutoff scores have 86% sensitivity and 72% specificity for identifying hazardous drinking and/or active alcohol use disorders in men, and 66% sensitivity and 94% specificity likewise in women (37). AUDIT-C scores were dichotomized separately for males and females using these cutoff scores for the presence or absence of hazardous drinking.

#### 2.2.5. Perceived stress

The Perceived Stress Scale-10 (PSS-10) was used to assess stress in the past 30 days (38). The PSS-10 has been validated in the general population, adults, college and graduate students, and in clinical populations (39). Scores range from 0 to 40, with higher scores indicating higher perceived stress. Scores <13 indicate low perceived stress, 14–26 indicate moderate perceived stress, and ≥27 indicate high perceived stress (39). PSS-10 scores were categorized using these thresholds.

#### 2.2.6. Financial distress

Three questions measured financial distress and were adapted from the Center for Disease Control and Prevention's Behavioral Risk Factor Surveillance System Questionnaire (40): (1) “In general, would you say you have more money than you need, just enough for your needs, or not enough to meet your needs?” (2) “How difficult is it to pay your monthly bills—very difficult, somewhat, not very, or not at all difficult?” and (3) “In the past 12 months did you not have enough money to buy food—often, sometimes, rarely, or never?” Due to the distribution of responses, these variables were dichotomized; the financial need was coded as “yes” if the participant selected the “not enough” category, bill payment difficulty was coded as “yes” if the participant selected “very” or “somewhat,” and food insecurity was coded as “yes” if the participant selected “often” or “sometimes.”

#### 2.2.7. Impostor syndrome

The Clance Imposter Syndrome (CIS) scale was used to assess the extent to which participants feel their accomplishments are due to luck or chance instead of ability and the perceived feeling of impostorism (41). The CIS has been validated for use in clinical and general population samples including student populations (42). Scores range between 20 and 100 with scores <40 classified as few impostor characteristics (IC), 41–60 as moderate IC, 61–80 as frequent IC, and >80 as intense IC (42, 43). Sum scores were categorized using these thresholds.

#### 2.2.8. Covariates

Participant biological sex (44, 45), year in medical school (12), race, ethnicity (46), and family history of depression or suicide (depending on the model) were included as covariates in models examining the adjusted relations between mental distress indicators and risk factors. Questions about family history were adapted from the Center for Disease Control and Prevention's Behavioral Risk Factor Surveillance System Questionnaire (40).

### 2.3. Statistical analysis

**Figure 1:**
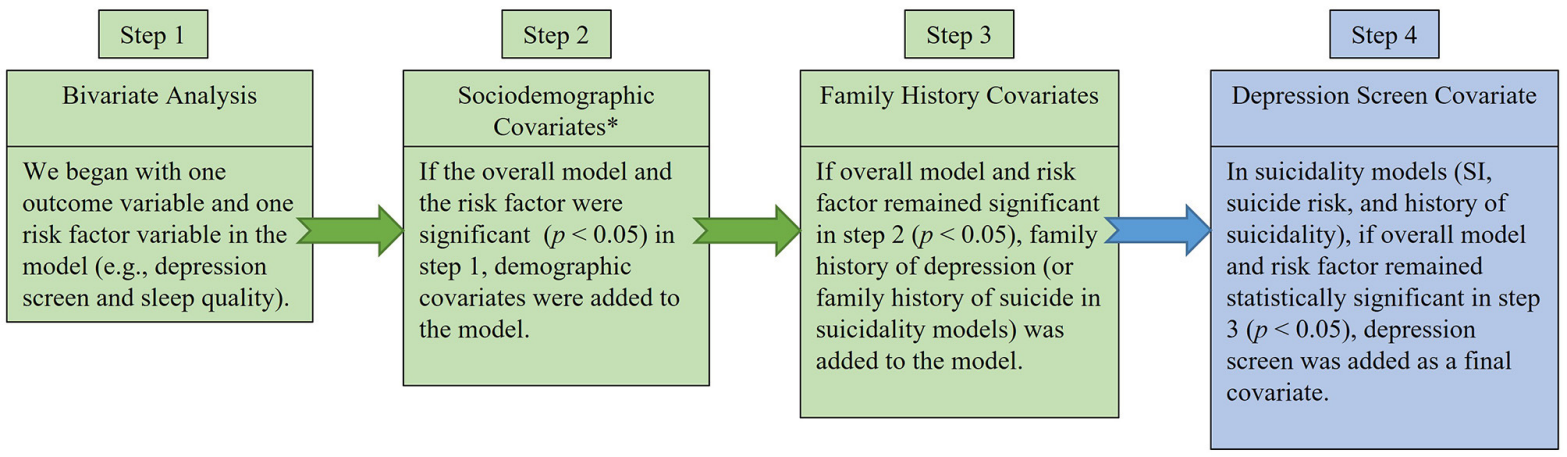
Logistic regression flow chart. Purpose of step 1 was to determine if the outcome variable (i.e., depression screen, suicidal ideation in the previous 12 months, suicide risk, and history of suicidality) was statistically significantly associated with each risk factor (i.e., sleep quality, alcohol use, stress, impostor-feelings, difficulty paying bills, financial need, and food insecurity). The purpose of step 2 was to control for *sociodemographic characteristics including sex, race, ethnicity, age, and year in medical school. The purpose of step 3 was to determine if the association between the mental distress outcome and risk factor remained statistically significant even after controlling for a family history of the disease. The purpose of step 4 was to determine if the risk factor was statistically significantly associated with the suicidality outcome, independently of the depression screen.

Descriptive statistics were used to characterize the sample, and chi-squared tests compared mental distress indicators and mental distress risk factors across biological sex. To examine associations between risk factors and mental distress indices, a sequence of logistic regression models was conducted (Figure 1). Separate logistic regression models examined relations between each risk factor and each dichotomized mental distress indicator (DS, SI in the previous 12 months, history of suicidality, and overall suicide risk score). Covariates were added in blocks to assess adjusted associations (Figure 1), and after full adjustment, predicted probabilities were computed and plotted for each model to visualize the marginal effects across mental distress groups [i.e., positive vs. negative depressive screen (DS)] (47).

Model robustness was verified for all logistic regression models through post-estimation analyses examining the linearity of odds, specification error, multicollinearity of independent variables, goodness of fit, and area under ROC curve. Post-estimation analysis included sensitivity analyses based on residuals, leverage, and influence diagnostics to ensure that individual observations were not driving the results. A Bonferroni correction was calculated to adjust for multiple comparisons. There were seven risk factor variables examined in this study, thus α^1^ = α/7 = 0.05/7 = 0.007. Statistical analyses were performed using Stata/SE 15.1.

## 3. Results

### 3.1. Mental distress indicators and risk factors among male and female medical students

Four-hundred and seventeen students were invited to participate, and 134 students enrolled in and completed the study (i.e., 32% of invited students enrolled in the study). Table 1 provides sample sociodemographic characteristics. Table 2 presents mental distress and risk factor data. There were no statistically significant biological sex differences for any mental distress indicator. Half of the sample screened positive for hazardous drinking, and the majority of participants reported poor quality sleep and at least moderate perceived stress. Nearly half the students reported frequent/intense impostor feelings. The only statistically significant difference in risk factors by biological sex was for hazardous drinking with females more likely than males to have a positive screen [$\chi_{\left(1,\text{N}=134\right)}^{2}$ = 9.6, *p* = 0.002].

**Table 1:** Sample sociodemographic characteristics (*N* = 134).

		***n* (%)**
Year in school	First	42 (31.34)
	Second	24 (17.91)
	Third	31 (23.13)
	Fourth	37 (27.61)
		***n*** **(%)**
Sex	Female	70 (52.24)
	Male	64 (46.76)
	**Mean (standard deviation)**	**Range**
Age (years)	25.75 (2.3)	22–36
		***n*** **(%)**
Race^*^	Asian	62 (46.27)
	White	59 (44.03)
	Black	3 (2.24)
	American Indian/Alaskan Native	1 (0.75)
	Native Hawaiian/Pacific Islander	1 (0.75)
		***n*** **(%)**
Hispanic/Latino	Yes	23 (17.16)

There was no missing data for any of the variables listed in this table. ^*^n = 8 (5.97%) of participants answered “Prefer not to answer.” These participants were added to an “other” group in analyses, with the n = 1 participant who answered “American Indian/Alaskan Native” and the n = 1 participant who answered “Native Hawaiian/Pacific Islander.” These participants' data were considered conceptually important to the results, even if they chose not to disclose their race. Thus, there was no missing data for any variables in this table. Age was used as a continuous variable in analyses.

**Table 2:** Sample characteristics—mental distress indicators and risk factors (*N* = 134).

		**Total**	**Males**	**Females**
		***n*** **(%)**		
Depression screen	Positive	22 (16.42)	8 (12.50)	14 (20.0)
	Negative	112 (83.58)	56 (87.5)	56 (80.0)
Depressive symptom severity^a^	Minimal or none	79 (58.96)	40 (62.50)	39 (55.71)
	Mild	33 (24.63)	16 (25)	17 (24.29)
	Moderate to moderately severe	22 (16.42)	8 (12.50)	14 (20)
Family history of depression diagnosis^b^	Yes	49 (36.57)	22 (34.38)	27 (38.57)
	No	66 (49.25)	34 (53.13)	32 (45.71)
Suicidal ideation in the previous 12 months	Yes	23 (17.16)	14 (21.88)	9 (12.86)
	No	111 (82.84)	50 (78.12)	61 (87.14)
History of suicidality	Yes	46 (34.33)	23 (35.94)	23 (32.86)
	No	88 (65.67)	41 (58.57)	47 (67.14)
Suicide risk screen	Positive	14 (10.45)	8 (12.50)	6 (8.57)
	Negative	120 (89.55)	56 (87.50)	64 (91.43)
Family history of suicide^c^	Yes	17 (12.69)	7 (10.94)	10 (14.29)
	No	116 (86.57)	57 (89.06)	59 (84.29)
Hazardous drinking screen	Positive	67 (50)	23 (35.94)	44 (62.86)
	Negative	67 (50)	41 (64.06)	26 (37.14)
Sleep quality	Good	55 (41.04)	28 (43.75)	27 (38.57)
	Poor	79 (58.96)	36 (56.25)	43 (61.43)
Perceived stress	Low	60 (44.78)	39 (60.94)	21 (30.0)
	Moderate	68 (50.75)	21 (32.81)	47 (67.14)
	High	6 (4.48)	4 (6.25)	2 (2.86)
Impostor feelings	Few	7 (5.22)	4 (6.25)	3 (4.29)
	Moderate	63 (47.01)	35 (54.69)	28 (40.0)
	Frequent	45 (33.58)	16 (25.0)	29 (41.43)
	Intense	19 (14.18)	9 (14.06)	10 (14.29)
Bill payment difficulty	Yes	33 (25)	14 (22.22)	19 (27.54)
	No	99 (75)	49 (77.78)	50 (72.46)
Financial need	Yes	23 (17.29)	10 (15.62)	13 (18.84)
	No	110 (82.71)	54 (84.37)	56 (81.15)
Food insecurity	Yes	19 (14.18)	11 (17.19)	8 (11.43)
	No	115 (85.82)	53 (82.81)	62 (88.57)

Depression screen and depressive symptom severity measured by Patient Health Questionnaire-9. Suicidal ideation in the previous 12 months, history of suicidality, and suicide risk measured by the Suicidal Behaviors Questionnaire-Revised. Hazardous drinking screen measured by Alcohol Use Disorders Identification Test-C. Sleep quality is measured by Pittsburgh Sleep Quality Index. Perceived Stress measured by Perceived Stress Scale-10. Impostor feelings are measured by the Clance Impostor Syndrome scale.

^a^Scores of 5, 10, 15, and 20 on the PHQ-9 correspond to mild, moderate, moderately severe, and severe depressive symptoms, respectively.

^b^n = 19 (14.18%) participants answered “I don't know” and were considered as missing in analyses.

^c^n = 1 (0.75%) participants answered “I don't know” and were considered as missing in analyses. There was no missing data for any other variables in this table.

### 3.2. Risk factors associated with a positive DS

**Figure 2:**
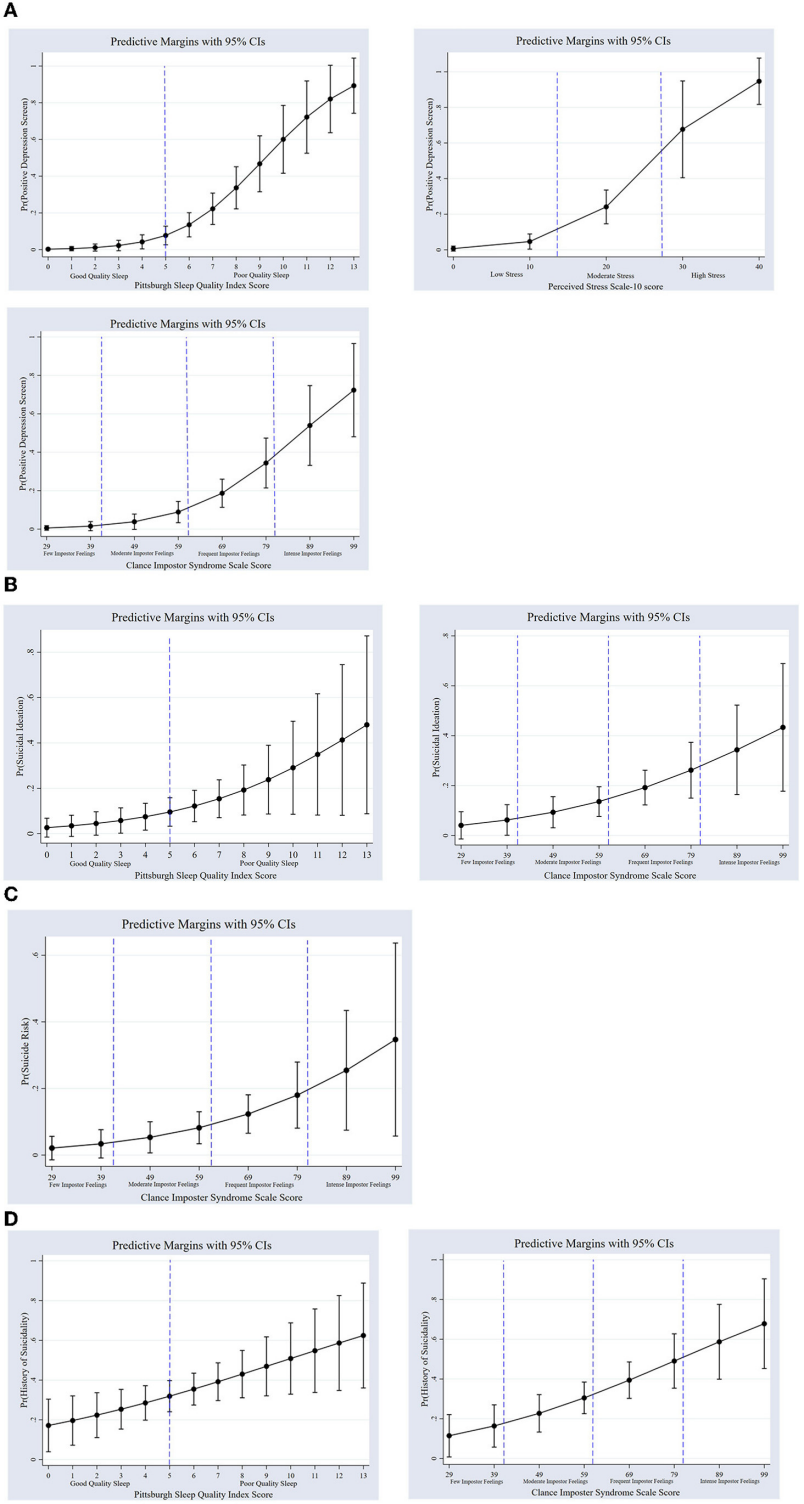
**(A)** Predicted probabilities of a positive depression screen by sleep quality, stress, and impostor feelings among medical students (*n* = 115). Predicted probabilities are from fully adjusted (up to step 3 in Figure 1) models. Dashed lines refer to cutoffs indicating (a) poor sleep quality, (b) moderate stress (14–26) and high stress (≥27), and (c) moderate impostor feelings (41–60), frequent impostor feelings (61–80), and intense impostor feelings (≥81). Depression screen and depressive symptom severity measured by Patient Health Questionnaire-9. Sleep quality is measured by Pittsburgh Sleep Quality Index. Perceived Stress is measured by Perceived Stress Scale-10. Impostor feelings are measured by the Clance Impostor Syndrome scale. **(B)** Predicted probabilities of suicidal ideation by sleep quality and impostor feelings among medical students (*n* = 133). Dashed lines refer to cutoffs indicating (a) poor sleep quality, and (b) moderate impostor feelings (41–60), frequent impostor feelings (61–80), and intense impostor feelings (≥81). Suicidal ideation in the previous 12 months measured by Suicidal Behaviors Questionnaire-Revised. Sleep quality is measured by Pittsburgh Sleep Quality Index. Impostor feelings are measured by the Clance Impostor Syndrome scale. **(C)** Predicted probabilities of suicide risk by impostor feelings among medical students (*n* = 134). Dashed lines refer to cutoffs indicating moderate impostor feelings (41–60), frequent impostor feelings (61–80), and intense impostor feelings (≥81). Suicide risk measured by the Suicidal Behaviors Questionnaire-Revised. Impostor feelings are measured by the Clance Impostor Syndrome scale. **(D)** Predicted probabilities of suicidal history by sleep quality and impostor feelings among medical students (*n* = 133). Dashed lines refer to cutoffs indicating (a) poor sleep quality and (b) moderate impostor feelings (41–60), frequent impostor feelings (61–80), and intense impostor feelings (≥81). History of suicidality measured by the Suicidal Behaviors Questionnaire-Revised. Sleep quality is measured by Pittsburgh Sleep Quality Index. Impostor feelings are measured by the Clance Impostor Syndrome scale.

After adjusting for all covariates, poor sleep quality, higher perceived stress, higher levels of imposter feelings, and difficulty paying bills were associated with increased odds of having a positive DS (Table 3A). Among scores indicative of poor sleep quality (≥5), the predicted probability of having a positive DS increased from 8.5% at a score of 5 [*p* = 0.004, 95% CI (0.027–0.142)], to 85.3% [*p* = 0.000, 95% CI (0.655–1.05)] at a sleep quality score of 13 (the highest reported in the sample; Figure 2A; panel a). As perceived stress scores increased from moderate to high, the predicted probability of having a positive DS increased from 24.1% [*p* = 0.000, 95% CI (0.146–0.335)] to 94.6% [*p* = 0.000, 95% CI (0.816–1.076); Figure 2A; panel b]. As impostor feelings increased from moderate to intense, the predicted probability of having a positive DS increased from 9.24% [*p* = 0.003, 95% CI (0.032–0.152)] to 70.56% [*p* = 0.000, 95% CI (0.446–0.964); Figure 2A; panel c]. The predicted probability of having a positive DS increased from 11.8% [*p* = 0.000, 95% CI (0.052–0.183)] for those who reported no difficulty paying bills, to 28.8% [*p* = 0.000, 95% CI (0.135–0.441)] for those who reported difficulty paying bills. Financial need [OR = 3, *p* = 0.040, 95% CI (1.049–8.576)] and food insecurity [OR = 3.88, *p* = 0014, 95% CI (1.322–11.435)] were also associated with increased odds of having a positive DS in bivariate analyses, but not after adjustment for covariates. A hazardous drinking screen was not associated with the odds of having a positive DS. The effects of sleep quality, perceived stress, and impostor feelings on the odds of a positive DS remained statistically significant at Bonferroni-adjusted alpha levels, while bill payment difficulty did not.

**Table 3A:** Adjusted associations between behavioral and psychosocial risk factors and odds of a positive depression screen among medical students (*N* = 134).

	**Odds ratio**	***p***	**95% CI**
**Sleep quality model (*****n*** = **115)**			
Sleep quality	2.02	**0.000** ^*^	1.430–2.867
Female	3.03	0.168	0.626–14.702
Race: White	0.68	0.661	0.129–3.651
Race: Other	0.19	0.242	0.012–3.002
Hispanic/Latino	3.58	0.190	0.531–24.249
Year in school: Second	0.72	0.751	0.098–5.325
Year in school: Third	0.15	0.119	0.015–1.604
Year in school: Fourth	0.03	**0.016**	0.001–0.519
Age	1.30	0.128	0.914–2.030
Family history^a, b^	0.77	0.727	0.184–3.254
**Perceived stress model (*****n*** = **115)**			
Stress	1.268	**0.000** ^*^	1.11–1.44
Female	1.51	0.570	0.362–6.327
Race: White	0.60	0.545	0.118–3.088
Race: Other	0.24	0.274	0.019–3.037
Hispanic/Latino	6.49	**0.039**	1.101–38.256
Year in school: Second	0.79	0.784	0.147–4.241
Year in school: Third	0.15	0.074	0.020–1.201
Year in school: Fourth	0.05	0.051	0.007–1.010
Age	1.40	0.061	0.985–1.993
Family history^a, b^	1.42	0.602	0.377–5.363
**Impostor feelings model (*****n*** = **115)**			
Impostor feelings	1.10	**0.000** ^*^	1.047–1.174
Female	1.21	0.784	0.300–4.911
Race: White	1.83	0.484	0.335–9.995
Race: Other	0.62	0.714	0.049–7.799
Hispanic/Latino	6.52	**0.031**	1.184–35.955
Year in school: Second	1.16	0.870	0.194–6.935
Year in school: Third	0.22	0.170	0.027–1.886
Year in school: Fourth	0.11	0.061	0.011–1.103
Age	1.08	0.641	0.776–1.508
Family history^a, b^	1.22	0.759	0.337–4.443
**Bill payment difficulty model (*****n*** = **115)**			
Bill difficulty	3.43	**0.046**	1.02–11.5
Female	1.39	0.563	0.454–4.25
Race: White	0.549	0.359	0.152–1.97
Race: Other	0.177	0.158	0.016–1.95
Hispanic/Latino	3.61	0.099	0.786–16.6
Year in school: Second	0.628	0.561	0.131–3.00
Year in school: Third	0.407	0.269	0.082–2.00
Year in school: Fourth	0.097	**0.025**	0.012–0.745
Age	1.209	0.215	0.895–1.634
Family history^a, b^	1.387	0.568	0.451–4.26

The results presented in this table are from four separate logistic regression models: sleep quality, stress, impostor feelings, and bill payment difficulty. The table presents results from step 3 in the logistic regression flow chart (see Figure 1), where family history of depression was added to each model.

^*^After Bonferroni correction for multiple comparisons (0.05/7), the new p-value = 0.007. Of the main predictors in the above models, sleep quality, stress, and impostor feelings are robust to this correction. Bill payment difficulty is not.

^a^Family history refers to the family history of depression.

^b^n = 19 (14.18%) participants answered “I don't know” and were considered as missing in analyses.

Bold values correspond to statistically significant covariates.

### 3.3. Risk factors associated with SI

Sleep quality was associated with higher odds of having SI after adjusting for all covariates, including DS (Table 3B). However, sensitivity analyses revealed that one participant was likely driving the association between SI and sleep quality. When this participant was dropped from the sleep quality model, the statistical significance of the sleep quality variable changed from *p* = 0.047 to *p* = 0.166. This participant had a high-risk profile with a positive DS, SI in the previous 12 months, and the highest (poorest) sleep quality score in this sample.

**Table 3B:** Adjusted associations between behavioral and psychosocial risk factors and odds of suicidal ideation among medical students (*N* = 134).

	**Adjustment for family history of suicide**			**Adjustment for depression screen**		
	**Odds ratio**	***p***	**95% CI**	**Odds ratio**	***p***	**95% CI**
**Sleep quality model (*****n*** = **133)**						
Sleep quality	1.30	**0.019**	1.04–1.62	1.27	**0.047**	1.00–1.64
Female	0.25	**0.023**	0.08–0.82	0.25	**0.022**	0.07–0.81
Race: White	0.58	0.419	0.16–2.13	0.58	0.412	0.13–1.81
Race: Other	2.68	0.261	0.47–14.99	2.73	0.252	0.48–15.35
Hispanic/Latino	1.76	0.471	0.37–8.32	1.76	0.476	0.37–8.36
Year in school: Second	0.09	0.056	0.01–1.05	0.09	0.055	0.01–1.05
Year in school: Third	0.90	0.896	0.21–3.80	0.92	0.916	0.22–3.89
Year in school: Fourth	0.46	0.390	0.08–2.66	0.49	0.429	0.08–2.86
Age	0.98	0.929	0.71–1.35	0.98	0.904	0.71–1.34
Family history^a, b^	1.01	0.981	0.22–4.52	1.03	0.968	0.23–4.55
Depression screen				1.30	0.732	0.28–5.95
**Impostor feelings model (*****n*** = **133)**						
Impostor feelings	1.05	**0.011**	1.01–1.09	1.05	**0.024**	1.01–1.09
Female	0.23	**0.016**	0.07–0.75	0.23	**0.015**	0.07–0.75
Race: White	0.65	0.537	0.16–2.56	0.64	0.523	0.16–2.54
Race: Other	1.52	0.640	0.23–10.81	1.52	0.668	0.22–10.54
Hispanic/Latino	4.06	0.077	0.85–19.19	3.95	0.087	0.82–19.16
Year in school: Second	0.10	0.053	0.01−1.03	0.09	0.052	0.01–1.01
Year in school: Third	0.66	0.565	0.16–2.70	0.66	0.565	0.16–2.74
Year in school: Fourth	0.29	0.174	0.05–1.71	0.29	0.189	0.05–1.82
Age	0.93	0.677	0.69–1.27	0.93	0.686	0.69–1.27
Family history^a, b^	1.35	0.693	0.30–6.03	1.36	0.685	0.30–6.04
Depression screen				1.18	0.816	0.29–4.78

The results presented in this table are from two separate logistic regression models: sleep quality and impostor feelings. The table presents results from step 3 (i.e., adjustment for family history of suicide), and step 4 (i.e., adjustment for depression screen) of the logistic regression flow chart, as seen in Figure 1. After Bonferroni correction for multiple comparisons (0.05/7), the new p-value = 0.007. Of the main predictors in the above models, sleep quality and impostor feelings are not robust to this correction.

^a^Family history refers to the family history of suicide.

^b^n = 1 (0.075%) participant answered “I don't know” and was considered as missing in analyses.

Bold values correspond to statistically significant covariates.

Adjusting for all covariates, higher levels of impostor feelings were also associated with increased odds of having SI (Table 3B). This association remained significant even after controlling for DS (Table 3B). As impostor feelings scores increased from moderate to intense, the predicted probability of having SI increased from 10.4% [*p* = 0.002, 95% CI (0.037–0.170)] to 42.7% [*p* = 0.002, 95% CI (0.162–0.692); Figure 2B; panel b]. Stress, bill payment difficulty, financial need, food insecurity, and hazardous drinking screen were not associated with increased odds of having SI. Neither the effects of sleep quality nor impostor feelings on the odds of having SI remained statistically significant after Bonferroni adjustments of alpha levels.

### 3.4. Risk factors associated with suicide risk

Higher levels of impostor feelings were associated with an increased odds of having a positive suicide risk screen after adjustment for sociodemographic characteristics (i.e., step 2 in Figure 1; Table 3C). When proceeding to step 3 in the logistic regression flow chart, where we control for family history of suicide, this variable was automatically dropped from the model because none of the *n* = 14 (10.45%) individuals who screened positive for suicide risk had a family history of suicide. When the individuals with a family history of suicide were excluded from the step 3 model, the results remained the same. When controlling for DS (i.e., step 4 in Figure 1), the association between higher imposter feelings and a positive suicide risk screen remained statistically significant (Table 3C). As the impostor feelings scores increased from moderate to intense, the predicted probability of having a positive suicide risk screen increased from 5.1% [*p* = 0.030, 95% CI (0.004–0.096)] to 38.2% [*p* = 0.022, 95% CI (0.055–0.708); Figure 2C]. Poorer sleep quality [OR = 1.24, *p* = 0.039, 95% CI (1.010–1.526)] and higher perceived stress [OR = 1.10, *p* = 0.031, 95% CI (1.01–1.201)] were also associated with increased odds of having a positive suicide risk screen in bivariate analysis, but not after adjustment for covariates. Hazardous drinking screen, bill payment difficulty, financial need, and food insecurity were not associated with the odds of having a positive suicide risk screen. In the step 4 model with the family history of suicide covariate removed, the effects of impostor feelings on the odds of having a positive suicide risk screen did not remain statistically significant after a Bonferroni adjustment of alpha levels.

**Table 3C:** Adjusted associations between behavioral and psychosocial risk factors and odds of suicide risk among medical students (*N* = 134).

	**Adjustment for sociodemographic characteristics**			**Adjustment for depression screen**		
	**Odds ratio**	***p***	**95% CI**	**Odds ratio**	***p***	**95% CI**
**Impostor feelings model (*****n*** = **134)**^a^						
Impostor feelings	1.05	**0.021**	1.01–1.10	1.06	**0.020**	1.01–1.11
Female	0.36	0.163	0.08–1.51	0.35	0.163	0.08–1.51
Race: White	0.80	0.800	0.14–4.44	0.80	0.805	0.14–4.48
Race: Other	9.05	**0.031**	1.21–67.40	8.80	**0.034**	1.18–66.07
Hispanic/Latino	1.75	0.522	0.31–9.72	1.95	0.454	0.33–11.36
Year in school: Second	1.47	0.716	0.18–11.94	1.55	0.681	0.08–1.51
Year in school: Third	2.78	0.263	0.46–16.70	2.63	0.291	0.43–15.98
Year in school: Fourth	0.99	0.997	0.09–10.31	0.87	0.911	0.07–9.59
Age	0.94	0.746	0.64–1.36	0.94	0.789	0.65–1.38
Depression screen				0.60	0.585	0.10–3.63

The table presents results from step 2 (i.e., adjustment for sociodemographic characteristics) and step 4 (i.e., adjustment for depression screen) of the logistic regression flow chart, as seen in Figure 1.

^a^Family history of suicide was ultimately dropped from these models because none of the n = 14 (10.45%) individuals who screened positive for suicide risk had a family history of suicide. After Bonferroni correction for multiple comparisons (0.05/7), the new *p* value = 0.007. Of the main predictors in the above models, impostor feelings is not robust to this correction.

Bold values correspond to statistically significant covariates.

### 3.5. Risk factors associated with history of suicidality

Poorer sleep quality was associated with increased odds of having a history of suicidality, even after adjusting for all covariates and controlling for DS (Table 3D). Higher levels of impostor feelings were also associated with increased odds of having a history of suicidality, even after adjustment for all covariates and after controlling for DS (Table 3D). Among scores indicative of poor sleep quality (≥ 5), the predicted probability of having a suicidal history increased as sleep quality worsened (higher score) from 31.8% [*p* = 0.000, 95% CI (0.240–0.397)] at a score of 5 to 62.4% [*p* = 0.000, 95% CI (0.360–0.888)] at a score of 13 (the highest score in this sample; Figure 2D; panel a). As impostor feelings increased from moderate to intense, the predicted probability of having a suicidal history increased from 21.4% [*p* = 0.000, 95% CI (0.121–0.307)] to 72.3% [*p* = 0.000, 95% CI (0.500–0.947); Figure 2D; panel b]. Perceived stress was associated with increased odds of having a history of suicidality [OR = 1.07, *p* = 0.025, 95% CI (1.08–1.12)], but not after adjustment for covariates. Hazardous drinking, bill payment difficulty, financial need, and food insecurity were not associated with the odds of having a history of suicidality. The effects of impostor feelings on the odds of having a history of suicidality remained statistically significant at Bonferroni-adjusted alpha levels, while sleep quality did not.

**Table 3D:** Adjusted associations between behavioral and psychosocial risk factors and odds of suicidal history among medical students (*N* = 134).

	**Adjustment for family history of suicide**			**Adjustment for depression screen**		
	**Odds ratio**	***p***	**95% CI**	**Odds ratio**	***p***	**95% CI**
**Sleep quality model (*****n*** = **133)**						
Sleep quality	1.19	**0.032**	1.01–1.40	1.26	**0.019**	1.03–1.53
Female	0.54	0.155	0.23–1.25	0.57	0.187	0.24–1.31
Race: White	0.28	**0.009**	0.10–0.72	0.26	**0.007**	0.09–0.67
Race: Other	1.36	0.662	0.33–5.61	1.21	0.789	0.20–5.21
Hispanic/Latino	1.02	0.965	0.28–3.64	1.08	0.903	0.31–4.16
Year in school: Second	0.87	0.833	0.25–2.82	0.84	0.788	0.24–2.91
Year in school: Third	1.05	0.927	0.33–3.34	0.89	0.845	0.28–2.85
Year in school: Fourth	0.28	0.070	0.07–1.10	0.23	**0.047**	0.05–0.98
Age	1.09	0.443	0.87–1.36	1.12	0.321	0.89–1.41
Family history^a^	0.84	0.779	0.25–2.84	0.84	0.796	0.25–2.88
Depression screen				0.51	0.303	0.14–1.83
**Impostor feelings model (*****n*** = **133)**						
Impostor feelings	1.05	**0.003** ^*^	1.02–1.08	1.05	**0.001** ^*^	1.02–1.09
Female	0.51	0.131	0.22–1.22	0.46	0.080	0.19–1.09
Race: White	0.35	**0.038**	0.23–0.94	0.35	**0.037**	0.13–0.94
Race: Other	1.55	0.595	0.31–7.78	1.42	0.675	0.27–7.42
Hispanic/Latino	1.35	0.643	0.38–4.77	1.58	0.483	0.44–5.74
Year in school: Second	0.95	0.944	0.27–3.37	0.94	0.929	0.27–3.32
Year in school: Third	1.01	0.985	0.32–3.24	0.92	0.890	0.28–3.01
Year in school: Fourth	0.26	0.061	0.06–1.06	0.25	0.064	0.05–1.08
Age	1.01	0.923	0.80–1.27	1.03	0.796	0.81–1.31
Family history^a^	0.98	0.982	0.28–3.37	0.98	0.985	0.28–3.44
Depression screen				0.47	0.223	0.14–1.57

The results presented in the table are from two separate logistic regression models: sleep quality and impostor feelings. The table presents results from step 3 (i.e., adjustment for family history of suicide), and step 4 (i.e., adjustment for depression screen) of the logistic regression flow chart, as seen in Figure 1.

^a^Family history refers to family history of suicide.

^*^After Bonferroni correction for multiple comparisons (0.05/7), the new *p* value = 0.007. Of the main predictors in the above models, impostor feelings is robust to this correction. Sleep quality is not.

Bold values correspond to statistically significant covariates.

## 4. Discussion

This study provides novel and valuable insight into the prevalence and correlates of mental distress in the pre–COVID-19 era medical school population. The most salient risk factors identified in this study include sleep quality, impostor feelings, stress, and, to a lesser extent, financial distress. The prevalence of positive depression screens in this sample suggests a higher prevalence of depression than is typically reported in the general population but lower than medical students in previous studies (i.e., 27–58%) (6, 48). In contrast, the prevalence of SI in this sample is higher than is reported in both the general population and among medical students in previous studies (i.e., 9–11%) (6, 48). Additionally, approximately one-third of medical students in this sample had a history of suicidality (SI or suicide attempt), which may suggest that at-risk individuals are self-selecting into the medical field. This may partially explain the disparity in physician suicide relative to the general population. Alternatively, this could be an indication of how arduous the path to a career in medicine can be.

Impostor feelings emerged as a prominent risk factor for medical students, as high levels of imposter feelings were associated with increased odds of mental distress for every indicator in this study. These findings were robust to Bonferroni correction in the depression screen and history of suicidality models, suggesting very strong associations in this sample. With every 1-point increase in the impostor symptoms score (with higher scores indicative of higher levels of impostor feelings), the odds of having a positive depression screen increase by a factor of 1.10. In terms of suicidal history, with every 1-point increase in the impostor symptoms score, the odds of having a history of suicidality increase by a factor of 1.05. Although these odds ratios may appear relatively small, the overall effect is quite large given that the range of scores on the impostor syndrome scale is between 20 and 100. Individuals with impostor syndrome constantly doubt their skills and abilities, and are often fearful of being discovered as an impostor, or as someone who does not belong (49). This sample also had a very high prevalence of impostor feelings relative to other studies. A recent review of impostor syndrome in medical students reports prevalence among studies between 20 and 60% (26). Coping with feelings of impostor syndrome in medical students has not been explored in the literature. However, a study of the impostor phenomenon among academic faculty suggests potential intervention strategies for alleviating imposter feelings, such as formal institutional support and increasing the use of peer support networks (27). This is an area of future research with the potential to inform medical school-specific interventions.

As expected, a high proportion of students reported poor sleep quality and this was associated with increased odds of having a positive depression screen, SI in the previous 12 months, and a positive suicide risk screen (19, 20, 50). When adjusting for multiple comparisons, the effect of poor sleep on the odds of a positive depression screen remained statistically significant with a 1-point increase in sleep quality score (with higher scores indicative of worse sleep) corresponding to a 2.02-fold increase in odds of having a positive depression screen. These findings highlight the importance of improving sleep behaviors among medical students. This burden not only lies with the student but also extends to institutional levels and should be seriously considered during curriculum development and implementation. Stress was found to be another modifiable risk factor for depressive symptoms in our sample. Although much attention has been devoted toward the development of stress reduction programs for medical students [e.g., through mindfulness (51–53) and coping (54) interventions], these programs are not adopted by every institution. There are also mixed findings on the effectiveness of these interventions (51, 54–56). The results of this study support further examination of the mechanisms linking sleep and stress with depressive symptoms among medical students and how best to target intervention programs aimed at reducing depression.

A greater difficulty paying bills was associated with increased odds of having a positive depression screen in this sample, with individuals experiencing bill payment difficulty having a 3.43-fold increase in odds of having a positive depression screen relative to individuals who do not have difficulty paying bills. Although this statistically significant finding did not hold up to the conservative Bonferroni adjustment, the effect size of the odds ratio is so large that it warrants further discussion. Financial distress related to educational debt is associated with burnout in both medical students and resident physicians, and this is significant because burnout is associated with depression and SI in this population (7, 57, 58). This potential risk factor for mental distress may become more salient in the coming years for medical students as the American Association of Medical Colleges works toward expanding the physician workforce to include people from economically disadvantaged backgrounds (59). This means that the percentage of matriculants coming from the lower three quintiles of household income is likely to increase, which may lead to a greater percentage of medical students requiring loans to fund their medical education. This topic warrants further investigation, as the median educational debt for 73% of medical school graduates in 2019 was $200,000 (60). This figure increases yearly, even above what would be expected with inflation (60). Additional research is needed to investigate avenues to alleviate financial distress in medical students, as this is certainly a modifiable risk factor in this population (e.g., through grants, loan forgiveness, and reduction in the cost of medical school tuition).

Although hazardous alcohol use was not associated with a positive depression screen nor indicators of suicidality in this study, the fact that half of the sample screened positive for hazardous drinking is concerning. A nationwide study of physicians found that 12.9% of male physicians and 21.4% of female physicians screened positive for alcohol misuse (61). Although the prevalence was much higher in this study sample, the biological sex differences reported at the national level are also evident in this sample. The observed biological sex differences may be due to the lower threshold for hazardous drinking among females. Other studies report that high alcohol use is associated with depression, SI, low quality of life, recent medical errors, and suicide in physicians (61, 62). Our findings suggest that heavier alcohol consumption may be a symptom of underlying mental distress and co-occurring risk factors that begin early on in medical training, presenting an opportunity to mitigate this risk factor with supportive interventions.

### 4.1. Limitations

Although this study found high levels of suicidal ideation in this sample relative to other studies of medical students (6, 48), our sample was likely not large enough for statistically significant associations to remain so after adjustment for multiple comparisons. Additionally, we expected that a higher number of individuals would have high-risk profiles similar to the individual who was driving the sleep quality model (see Section 3.3). Our sense, based on the literature, is that a larger sample size including multiple institutions would address the issues of statistical significance. The sample size issue likely also comes into play when considering the impostor feelings and suicide risk model, where we could not control for family history of suicide because none of the individuals with a positive suicide risk screen had a family history of suicide. Although it created issues in our model building, this finding may suggest that one of the strongest predictors of suicide in the general population, family history of suicide (63), may not be as salient among medical students. That is to say, other factors are increasing the risk of suicide in this population, and thus we should focus on literature addressing these factors among medical students when building future studies. More research is needed to understand the construct of suicide risk among medical students. Despite the small sample size, we maintain that the associations observed in this study are conceptually important and warrant further investigation.

Due to the cross-sectional nature of this study, the findings cannot speak to the temporality of the development of depression and suicidality. Risk factors and mental health are often bi-directional and cyclical, and risk/resilience factors tend to occur in clusters. Thus, understanding relationships among risk factors, rather than studying independently, requires further analyses. These findings may also not be generalizable to all medical students as all participants came from one large medical school. However, it is important to note that our sample had greater racial and ethnic diversity than what is seen in medical students at the national level (64), thus findings may be more appropriately generalized to other highly diverse large medical schools.

Another limitation is that the study involved an in-person appointment with a member of the research team and participants completed the written survey in the study room. Given that mental health is more heavily stigmatized in this population, the data may be subject to a social desirability effect. It is important to note that all written responses in this study were anonymous to attempt to minimize this effect. However, any social desirability bias would only underestimate the findings of this study. Nevertheless, future studies should aim to further reduce the possibility of bias in the survey response to ensure participant comfort.

It is also important to mention that by the design of the questions on the Suicidal Behaviors Questionnaire-Revised and because of our coding method, there is an overlap among the SI and history of suicidality groups. Every individual who endorsed SI in the previous 12 months (*n* = 23), also has a history of suicidality (*n* = 46), so they are present in both groups. This overlapping of constructs makes it more difficult to tease apart factors related to the individual mental distress indicators. There was less overlap among individuals in the DS and SI (*n* = 6) groups and among individuals in the DS and history of suicidality (*n* = 9) groups. The overlap between mental distress indicators is not unexpected, given the hypothesized causal relationship between depression and suicidal thoughts and behaviors (65–67). We statistically controlled for this relationship in the suicidality models by adding the depression screen covariate to the logistic regression model as the fourth and final step in model building, as is detailed in Section 2.3.

### 4.2. Conclusion and implications

This study provides a comprehensive investigation into a broad range of risk factors for depression and suicidality in a pre–COVID-19 sample of medical students early in their training. It is also the first investigation to assess the prevalence of suicidal history in medical students and to consider financial distress during medical school as a potential risk factor for depression and suicidality. With new charges to recruit more diverse medical students in terms of race, ethnicity, and family socioeconomics, this finding is predicted to become more relevant with future cohorts. The findings support the importance of early screening and effective support during medical training. Longitudinal and qualitative studies are needed to determine at what point suicidality develops in (pre)medical students, and the contributing factors need to be further explored.

Our findings point to possible targets for individual and institutional-level interventions to support mental wellbeing among medical students, including mental distress screenings and programs aimed at improving sleep, reducing stress, addressing impostor syndrome, and reducing financial insecurity. Impostor syndrome stood out among all other risk factors in the present sample, thus more research is needed to understand potential mechanisms to inform intervention strategies. Longitudinal research is needed to examine the temporality of these relations to inform customized resources that support individuals at every stage of their medical training and career.

### 4.3. A way forward

It is important to acknowledge that the modification of individual-level risk factors is limited by the larger medical culture, stigma, and systemic factors. Successful interventions to mitigate suicide risk for medical providers need to address multiple socio-ecological levels. For example, future intervention studies exploring the effect of structural changes (i.e., reducing weekly mandatory class or clinic hours to promote healthy sleep behaviors and stress reduction) on mental distress in medical students are warranted.

The enhanced stigma of mental health issues is another larger cultural and systemic factor that may limit the impact of individual-level interventions. There is concern among physicians and medical students that seeking help for mental illness will jeopardize an individual's reputation, career prospects, and medical board licensure (12, 56, 68, 69). These concerns are well-founded because up until 2019, approximately two-thirds of states had medical licensure applications that asked questions about mental health in a way that violate the American Disabilities Act and the American Psychiatric Association's position that information about past mental health treatment is not a salient predictor of current impairment (70–73). For example, prior to April 2021, Florida's medical licensure application included yes/no questions that asked about mental health or substance use diagnoses, symptoms, and treatments within the past 5 years. If an applicant answered “yes” to these questions, the applicant was required to submit personal medical records, including information about diagnosis and treatment, to the medical board (73). These types of questions have resulted in physicians hiding their treatable mental health conditions, which has implications for not only the health and safety of the physician but also for their patients (56, 74). Within recent years, physicians have been lobbying for change at the state level, which has resulted in less discriminatory language on medical board applications in states such as New Mexico and Florida (73, 75). Currently, 21 states have invasive and inappropriate questions regarding mental health on their medical board licensure applications (72, 73). Although we are moving in a positive direction, more work is needed to protect the rights of physicians and medical students to receive care for treatable mental health conditions.

Personal stories of struggle and rebound are an incredibly powerful tool to fight mental health stigma within the medical community, and we are heartened to see stories being shared in the literature (74, 76–79). A recent commentary on physician suicide details the life and loss of Dr. Lorna Breen and shares stories from physicians who have attempted suicide and/or battled depression and suicidal thoughts (74). This willingness to share personal stories is called the lived experience movement, a movement that is “providing a hopeful arc to mental health experiences, shattering stigma, modeling help-seeking, and contributing to a new culture where mental health can be viewed and addressed openly and without shame” (74).

Finally, the larger community's and global health status is an active contextual factor that can act on these existing mechanisms and increase suicidality risk among the medical community. The present study's data collection was concluded months before the COVID-19 pandemic onset and does not reflect the predicted exacerbation by the global health context placing heightened demands on the medical field. Protecting the mental health of our nation's medical providers should be of utmost importance, given the implications it has for the health and safety of the general public. The disparities in depression, suicidal ideation, and suicide among physicians are clear, and we believe that the present study's findings contribute to the collective call for change.

## Data availability statement

The raw data supporting the conclusions of this article will be made available by the authors, without undue reservation.

## Ethics statement

The studies involving human participants were reviewed and approved by University of California, Irvine Office of Research Institutional Review Board. The patients/participants provided their written informed consent to participate in this study.

## Author contributions

KC collected the data, organized the database, and wrote the first draft of the manuscript. KC and HA performed the statistical analysis. KU and JR wrote sections of the manuscript. All authors contributed to conception, design of the study, interpretation of data, manuscript revision, read, and approved the submitted version.
